# Supplementary material for: Bayesian refinement of protein structures and ensembles against SAXS data using molecular dynamics
Source: PLoS Comput Biol. 2017 Oct 18;13(10):e1005800. doi: 10.1371/journal.pcbi.1005800 (PMC5662244; doi:10.1371/journal.pcbi.1005800)
Supplement: S1 Archive — (BZ2) [file pcbi.1005800.s011.tar.bz2 › gromacs_4.62_bayesian_saxs_refi/share/html/online/make_ndx.html]

make\_ndx


| make\_ndx Main Table of Contents |

**VERSION 4.6  
Sat 19 Jan 2013**

---

### Description

Index groups are necessary for almost every gromacs program.
All these programs can generate default index groups. You ONLY
have to use make\_ndx when you need SPECIAL index groups.
There is a default index group for the whole system, 9 default
index groups for proteins, and a default index group
is generated for every other residue name.

When no index file is supplied, also make\_ndx will generate the
default groups.
With the index editor you can select on atom, residue and chain names
and numbers.
When a run input file is supplied you can also select on atom type.
You can use NOT, AND and OR, you can split groups
into chains, residues or atoms. You can delete and rename groups.

The atom numbering in the editor and the index file starts at 1.

### Files

| option | filename | type | description |
| --- | --- | --- | --- |
| **-f** | conf.gro | Input, Opt. | Structure file: gro g96 pdb tpr etc. |
| **-n** | index.ndx | Input, Opt., Mult. | Index file |
| **-o** | index.ndx | Output | Index file |

### Other options

| option | type | default | description |
| --- | --- | --- | --- |
| **-[no]h** | bool | no | Print help info and quit || **-[no]version** | bool | no | Print version info and quit || **-nice** | int | 0 | Set the nicelevel || **-natoms** | int | 0 | set number of atoms (default: read from coordinate or index file) |

---

http://www.gromacs.org  
gromacs@gromacs.org
